# Long-term trends in the burden of multiple myeloma in China: a Joinpoint regression and age-period-cohort analysis based on GBD 2021

**DOI:** 10.3389/fpubh.2025.1554485

**Published:** 2025-02-12

**Authors:** Yanyu Zhang, Quanxin Su, Jiawen Yu, Xiuli Sun

**Affiliations:** ^1^Department of Hematology, The First Affiliated Hospital of Dalian Medical University, Dalian, China; ^2^Department of Urology, The First Affiliated Hospital of Dalian Medical University, Dalian, China

**Keywords:** multiple myeloma, burden of disease, Joinpoint regression analysis, age-period-cohort model, mortality

## Abstract

**Purpose:**

Multiple myeloma (MM) is a hematologic malignancy originating from plasma cells with clinical manifestations such as hypercalcemia, cytopenias (most commonly anemia) and renal failure. Here, we analyzed the disease burden and changing trends of MM in China from 1990 to 2021, aiming to provide a scientific and effective basis for the prevention and control of MM disease in China.

**Methods:**

We extracted MM related data from the Global Burden of Diseases (GBD) 2021 database from 1990 to 2021. It is described according to incidence, prevalence, mortality, disability-adjusted life years (DALYs), years lived with disability (YLDs), years of life lost (YLLs) and other indicators. Using Joinpoint regression model to analyze the long-term trends of disease burden of MM in China. Using the age-period-cohort (apc) model to analyze the impact of age, period, and birth cohort on the burden of MM.

**Results:**

It is estimated that in 2021, there were 17,250 new cases of MM in China, with 47,004 cases and 12,984 deaths. The age-standardized incidence (ASIR), prevalence (ASPR) and mortality rates (ASMR) per 100,000 people were 0.81 (95% CI: 0.52, 1.07), 2.19 (95% CI: 1.37, 2.90) and 0.62 (95% CI: 0.40, 0.81), respectively. A comparison of ASIR, ASPR and ASMR in 2021 with those in 1990 indicated an increase. The ASIR and ASMR of males are higher than those of females overall, and both were increasing with age. The ASIR exhibited a gradual upward trend, while ASPR (AAPC = 6.43, 95% CI: 5.90, 6.96) demonstrated the most substantial increase. The apc model indicated the net drift was found to be 3.70% (95%CI: 3.32, 4.08%) per year for incidence and 2.57% (95%CI: 2.24, 2.89%) per year for mortality. The effects of age, period, and cohort on the incidence and mortality rates exhibited significant variations. The incidence risk increased with age, but the mortality risk showed fluctuations.

**Conclusion:**

The trends of MM disease burden in China continued to increase from 1990 to 2021. MM will be a major challenge for the future healthcare sector in China, where the population base is large and gradually aging.

## Introduction

Multiple myeloma (MM) is the second most common malignancy of the lymphohematopoietic system (1), accounting for approximately 15% of all hematological tumors, and is essentially a plasma cell tumor characterized by an abnormal proliferation of monoclonal plasma cells and multi-focal skeletal involvement. Its manifestations can be summarized as CRAB symptoms, i.e., hypercalcemia, renal failure, anaemia or dissolving bone damage (2).

MM remains an incurable plasma cell malignancy. The incidence rate of MM is lowest in East Asia and Southeast Asia (3, 4) and occurs mainly in the older adult male population, with a median age of 69 for most diagnoses and about 10% of patients older than 85 years (5). In China, the incidence rate is 1.17/100000, with more males than females (6). Clinical manifestations at diagnosis are usually fatigue, anemia, bone pain and renal dysfunction. Due to the lack of specificity in the initial presentation, some patients with MM may be missed or misdiagnosed. Meanwhile, despite significant advances in the treatment of MM resulting in unprecedented survival rates, patients continue to relapse and a cure remains elusive. With the advent of immune-targeted therapies and immuno-modulators, the therapeutic agents and combination regimens available to MM patients are becoming increasingly diverse, and the prognosis for patients has improved significantly. However, the inability to achieve a fundamental cure for MM imposes a heavy and ongoing medical burden on patients, which has seriously affected the basic health status of the population. However, the data collection on MM patients in China is inadequate, and epidemiological reports on the disease are very limited.

Therefore, our study analyzed the epidemiological characteristics and developmental trends of MM based on the latest GBD 2021 data for China to provide a scientific and effective basis for the diagnosis and treatment of MM in China.

## Methods

### Data sources

The data for this study were drawn from the Global Burden of Disease 2021 (GBD 2021) study.^1^ The GBD2021 report encompasses 371 diseases and injuries and 88 risk factors in 204 countries and territories, and includes estimates for a wide range of disease and injury outcome models (7, 8).

### Joinpoint regression analysis

The Joinpoint regression models are a set of linear statistical models for trend analysis of change using the Joinpoint Regression Program 5.1.0 (National Cancer Institute, Washington, DC), which calculates average annual percentage change (AAPC) and annual percentage change (APC) from 1990 to 2021 using a logarithmic regression model. When APC > 0 and APC < 0, respectively, indicate an upward and downward trend during the period, while AAPC>0 and AAPC<0, respectively, indicate an upward and downward trend over time throughout the entire period; When AAPC = APC, it indicates a monotonic increasing or decreasing trend. The *p value < 0.05* was considered statistically significant.

### Age-period-cohort modeling analysis

Age-period-cohort (apc) models are utilized to evaluate the impact of age, period and birth cohort on outcomes. Utilizing the Poisson distribution, the target analytical variables are disaggregated by age, period and cohort, thereby facilitating enhanced analysis of the risk of incidence or mortality by age, period and cohort.

The following output parameters are derived from the age-period-cohort model:(1) Net drift: logarithmic linear trend of age standardization rate after adjusting for period and cohort effects;(2) Local drifts: logarithmic linear trend of age standardized rates for each age group after adjusting for period and cohort effects;(3) The longitudinal age curve: the specificity ratio of the longitudinal age curve fitted in the reference cohort after adjusting for temporal bias; (4) Rate ratio (RR) for the cohort (or period): relative risk adjusted for age and non-linear period effects relative to the reference period or cohort. Due to the linear relationship between age, period, and cohort, the endogenous estimator (IE) method can be used to avoid result bias. In the apc model, age and time interval data are unified because they need to be equal. In this study, age was appropriately reclassified into consecutive 5-year age groups (5–9, 10–14,…, and 90–94).The time range is from 1992 to 2021, divided into six consecutive 5-year periods (1992–1996 [1994], 1997–2001 [1999], 2002–2006 [2004],…, and 2017–2021 [2019]), as well as corresponding consecutive 5-year birth cohorts (1927–1932, 1932–1937, 1937–1942,…, and 2012–2017), totaling 18 cohorts, to estimate the net age, period, and cohort effects of MM. The significance of the function can be estimated through the Wald tests. Other analyses were conducted using the R Statistical Computing Program (version 4.4.1). All statistical tests were two-sided, and the *p value < 0.05* was considered statistically significant.

## Results

### Descriptive analysis

The incidence, incidence rate, mortality and mortality rate of MM in China from 1990 to 2021 were on the rise. In 1990, there were 1,693 cases (95% CI: 1154,3,360) of MM patients and 1,591 cases (95% CI: 1080,3,159) of deaths. However, by 2021, this number had increased to 17,250 cases (95% CI: 11,017, 22,663), with 12,984 deaths from MM (95% CI: 8,448, 17,114). In 2021, the number of cases had sharply increased, with a change rate of 9.19% (Supplementary Table S1). The incidence, mortality, and their rates of males in 2021 were higher than females. In 1990, the age-standardized incidence rate (ASIR), the age-standardized prevalence rate (ASPR), the age-standardized mortality rate (ASMR), the age-standardized disability-adjusted life years (DALYs), the age-standardized years lived with disability (YLDs), and the age-standardized years of life lost (YLLs) of MM were 0.81 (95% C: 0.52, 1.07), 2.19 (95% CI: 1.37, 2.90), 0.62 (95% CI: 0.40, 0.81), 16.12 (95% CI: 10.09, 21.35), 0.46 (95% CI: 0.28, 0.67), 15.66 (95% CI: 9.80, 20.70), respectively (Tables 1, 2).

**Table 1:** All-age cases and age-standardized prevalence, incidence, deaths, DALYs, YLDs, and YLLs rates in 1990 for multiple myeloma in China.

Measure	All-ages cases			Age-standardized rates per 100,000 people		
	Total	Male	Female	Total	Male	Female
Prevalence	2,977 (2,052, 5,851)	1,658 (1,076, 3,712)	1,319 (809, 3,169)	0.32 (0.22, 0.64)	0.37 (0.24, 0.84)	0.29 (0.17, 0.69)
Incidence	1,693 (1,154, 3,360)	927 (600, 2,106)	766 (462, 1869)	0.20 (0.13, 0.39)	0.23 (0.15, 0.52)	0.17 (0.10, 0.42)
Deaths	1,591 (1,080, 3,159)	858 (554, 1965)	733 (440, 1805)	0.19 (0.13, 0.39)	0.22 (0.14, 0.52)	0.17 (0.10, 0.42)
DALYs	46,854 (32,056, 92,593)	25,630 (16,776, 57,352)	21,224 (12,911, 50,908)	5.02 (3.42, 9.99)	5.59 (3.64, 12.77)	4.54 (2.74, 10.96)
YLDs	725 (400, 1,489)	405 (216, 883)	320 (159, 788)	0.08 (0.05, 0.17)	0.10 (0.05, 0.21)	0.07 (0.04, 0.18)
YLLs	46,129 (31,608, 91,267)	25,225 (16,459, 56,491)	20,904 (12,683, 50,250)	4.94 (3.36, 9.82)	5.49 (3.56, 12.56)	4.47 (2.69, 10.81)

DALYs, disability-adjusted life-years; YLDs, years lived with disability; YLLs, years of life lost.

**Table 2:** All-age cases and age-standardized prevalence, incidence, deaths, DALYs, YLDs, and YLLs rates in 2021 for multiple myeloma in China.

Measure	All-ages cases			Age-standardized rates per 100,000 people		
	Total	Male	Female	Total	Male	Female
Prevalence	47,004 (29,544, 62,136)	30,507 (16,633, 42,600)	16,497 (6,609, 23,984)	2.19 (1.37, 2.90)	2.90 (1.58, 4.06)	1.52 (0.60, 2.21)
Incidence	17,250 (11,017, 22,663)	10,772 (6,105, 15,153)	6,478 (2,716, 9,293)	0.81 (0.52, 1.07)	1.06 (0.60, 1.48)	0.59 (0.25, 0.84)
Deaths	12,984 (8,448, 17,114)	7,793 (4,530, 11,067)	5,191 (2,191, 7,400)	0.62 (0.40, 0.81)	0.79 (0.46, 1.11)	0.47 (0.20, 0.67)
DALYs	338,359 (213,669, 447,635)	204,807 (115,652, 287,644)	133,551 (55,689, 190,086)	16.12 (10.09, 21.35)	20.11 (11.29, 28.07)	12.40 (5.12, 17.76)
YLDs	10,036 (6,091,14,597)	6,533 (3,472,9,843)	3,502 (1,392,5,683)	0.46 (0.28,0.67)	0.63 (0.33,0.94)	0.32 (0.13,0.51)
YLLs	328,323 (207,346, 433,763)	198,274 (111,610, 277,663)	130,049 (54,276, 185,312)	15.66 (9.80, 20.70)	19.49 (10.90, 27.17)	12.09 (5.00, 17.27)

DALYs, disability-adjusted life-years; YLDs, years lived with disability; YLLs, years of life lost.

From 1990 to 2021, ASIR, ASPR, and ASMR of MM per 100,000 people in China changed from 0.20 (95% CI: 0.13, 0.39) to 0.81 (95% CI: 0.52, 1.07), from 0.32 (95% CI: 0.22, 0.64) to 2.19 (95% CI: 1.37, 2.90), and from 0.19 (95% CI: 0.13, 0.39) to 0.62 (95% CI: 0.40, 0.81). The maximum variation amplitude of ASPR was 584.38% (Supplementary Table S2). In contrast, the disease burden of males had always been higher than that of females.

**Figure 1:**
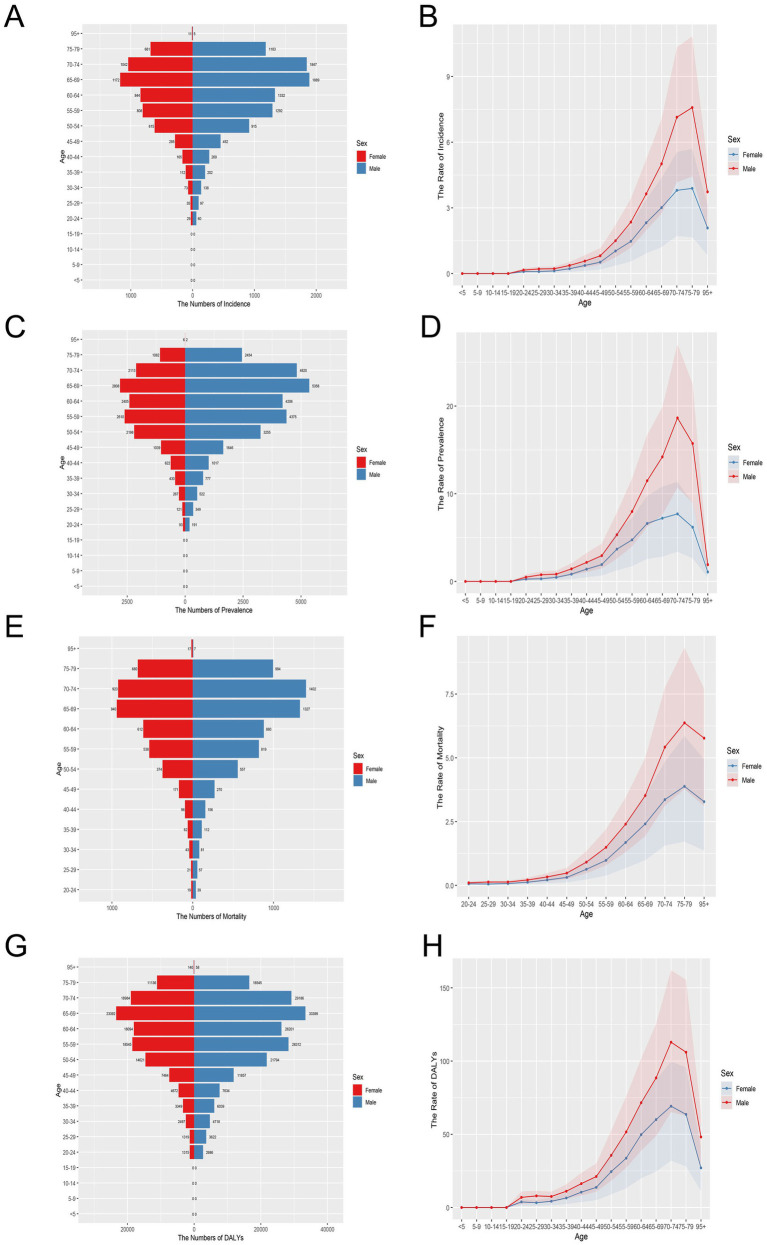
Age-specific numbers and age-standardized incidence, prevalence, mortality and DALYs rates of multiple myeloma in China, 2021. **(A)** Age-specific incidence number. **(B)** Age-standardized incidence rate. **(C)** Age-specific prevalence number. **(D)** Age-standardized prevalence rate. **(E)** Age-specific mortality number. **(F)** Age-standardized mortality rate. **(G)** Age-specific DALYs number. **(H)** Age-standardized DALYs rate.

It is noteworthy that the GBD 2021 database did not contain any data pertaining to MM patients under the age of 20. From the incidence results, the peak of incidence rate occurred in the 65–69 age group (Figures 1A,B). The increase of females incidence rate was greater. From the prevalence results, there was a brief fluctuation in 60–64 age group for both, with a decrease compared to 55–59 age group (Figures 1C,D). However, the peak of disease incidence for both males and females remained concentrated in 65–69 age group YLLs showed a similar trend (Supplementary Figure S1). From the perspective of mortality outcomes, the number of deaths in males was higher than that in females in all age groups, but the peak of deaths in females was between 65 and 69 age group, while the peak of deaths in males was between 70 and 74 age group (Figure 1E). The mortality rate increased sharply with age year by year (Figure 1F). There was no significant difference between the sexes. The age-standardized DALYs (ASDR), YLDs and ASIR in different gender and age groups showed similar trends (Supplementary Figure S1). The increase in DALYs was greater in males (Figures 1G,H). The population over 50 years old was a key group at high risk of MM.

**Figure 2:**
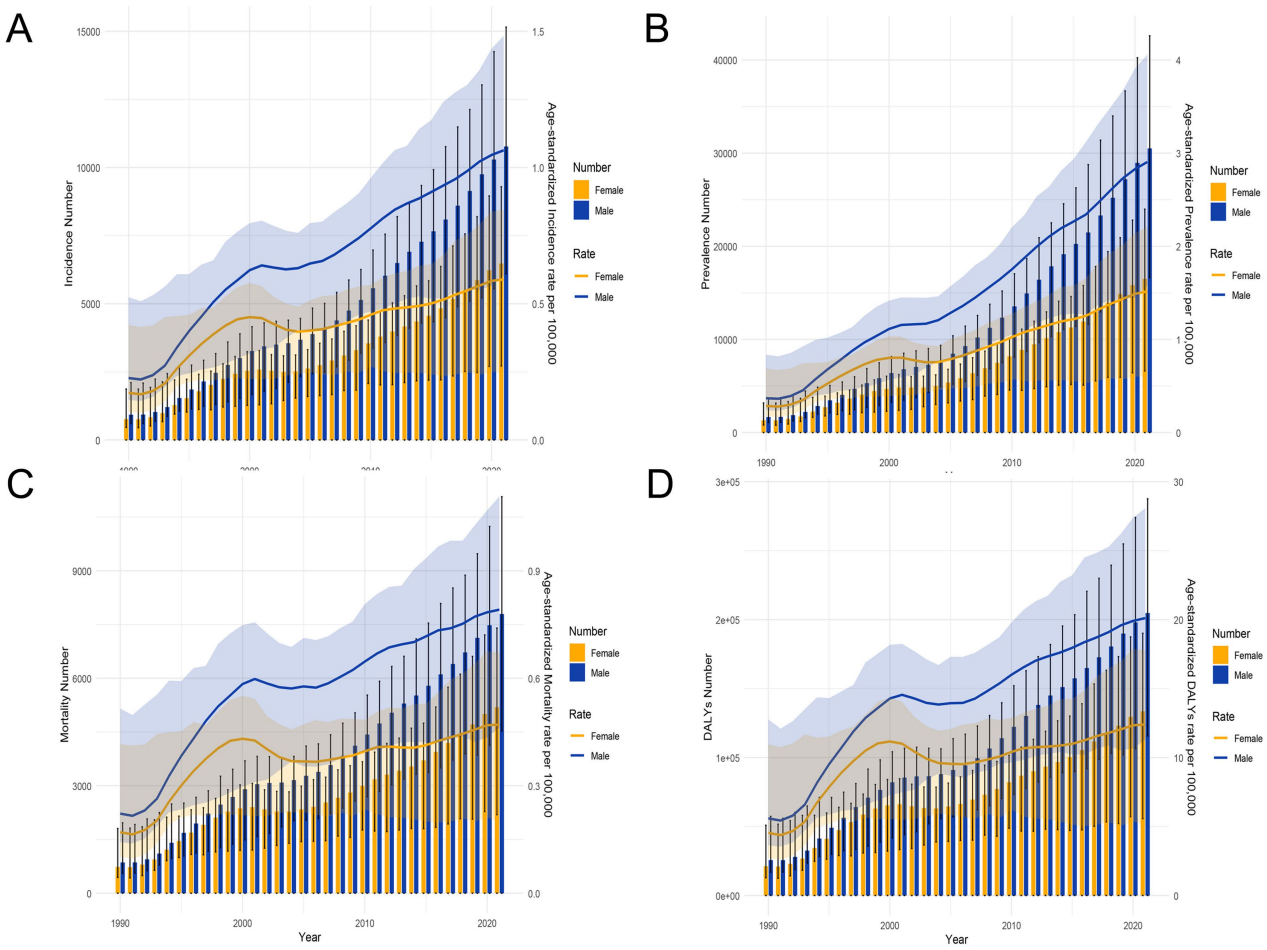
Trends in the all-age cases and age-standardized incidence, prevalence, mortality, and DALYs rates of multiple myeloma by sex from 1990 to 2021. **(A)** Incidence number and rate. **(B)** Prevalence number and rate. **(C)** Mortality number and rate. **(D)** DALYs number and rate.

Figure 2 illustrates the trend of changes in the age group and age-standardized rate of MM in males and females in China from 1990 to 2021. The incidence and mortality rates were generally on the rise. From 1990 to 2000, the incidence, mortality, DALYs and YLLs rates increased rapidly (Supplementary Figure S2). The gap between males and females was small, but after 2000, the gap gradually widened. The overall disease burden of males was relatively high. From 1990 to 2021, the incidence rates of both sexes showed a steady upward trend year by year. The results of YLDs were similar to theirs (Supplementary Figure S2).

### Joinpoint regression analysis

**Figure 3:**
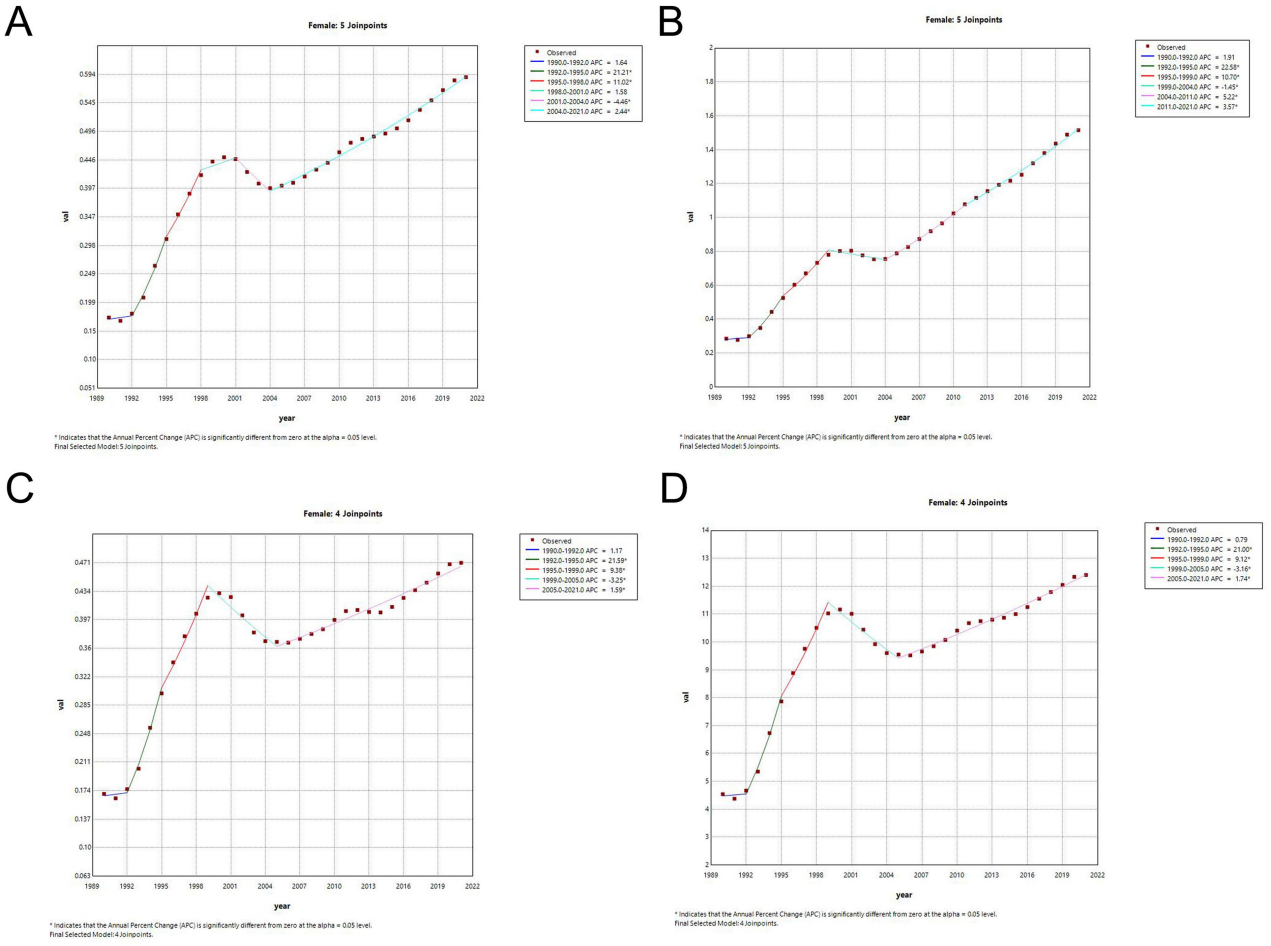
Joinpoint regression analysis of the sex-specific age-standardized rate for multiple myeloma in China from 1990–2021. **(A)** Age-standardized incidence rate for females. **(B)** Age-standardized prevalence rate for females. **(C)** Age-standardized mortality rate for females. **(D)** Age-standardized DALYs rate for females.

**Figure 4:**
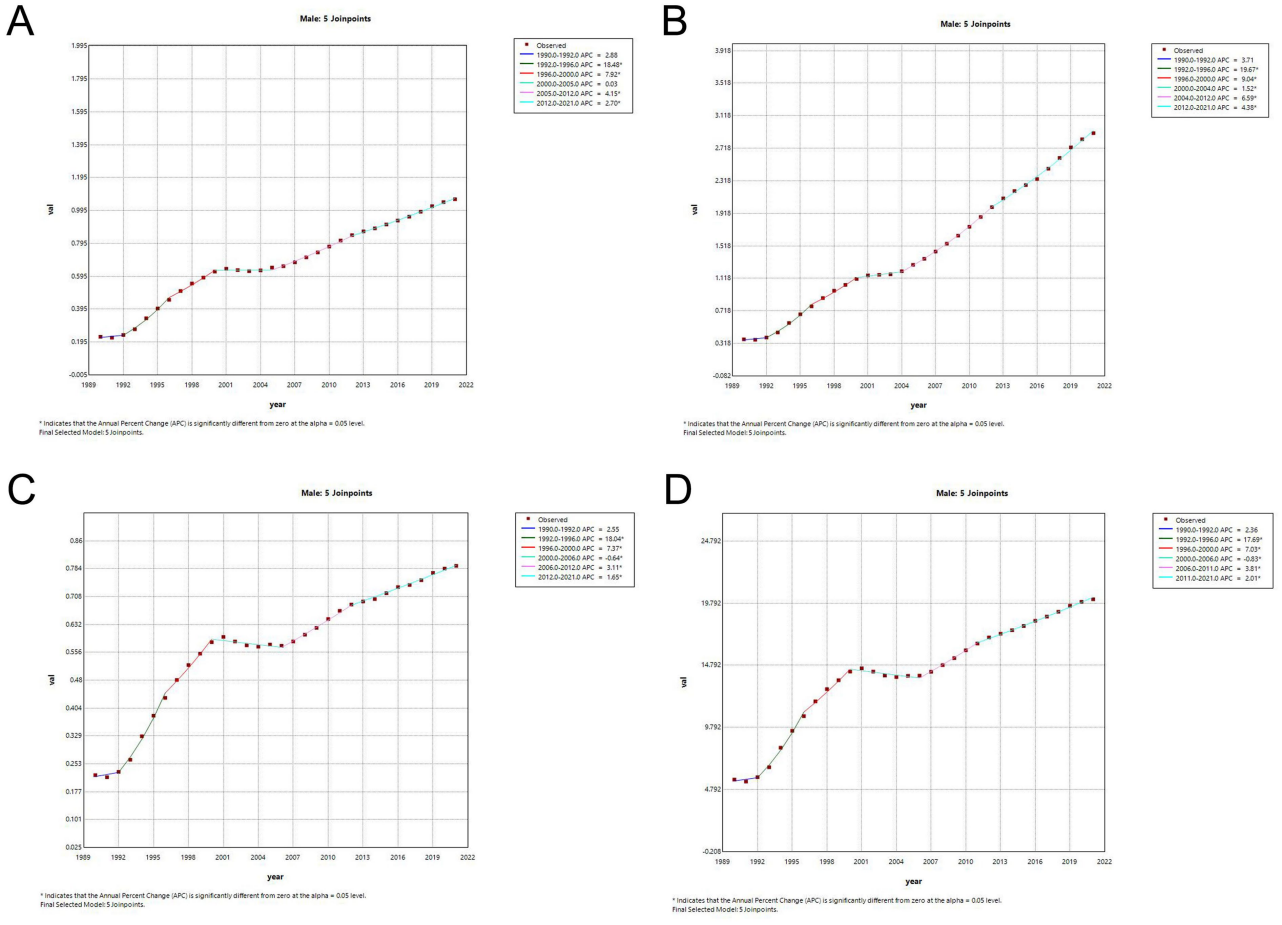
Joinpoint regression analysis of the sex-specific age-standardized rate for multiple myeloma in China from 1990–2021. **(A)** Age-standardized incidence rate for males. **(B)** Age-standardized prevalence rate for males. **(C)** Age-standardized mortality rate for females. **(D)** Age-standardized DALYs rate for males.

As illustrated in Table 3, the APC and AAPC of the incidence and mortality rates of MM in China from 1990 to 2021 are shown. The disease incidence and mortality trends of MM increased by 4.74 (AAPC, 95% CI: 4.18, 5.29) and 3.91 (AAPC, 95% CI: 2.79, 5.05), respectively. The incidence and DALYs trends for females was lower than that for males (Supplementary Table S3). Figures 3, 4 present the results of the Joinpoint regression analysis of age-standardized rates of MM in China from 1990–2021. The analysis revealed that from 1992 to 1995, the age-standardized rate of females increased by APC = +22.58 (95% CI: 17.51, 27.86) (Figure 3A), while the rate for males increased by APC = +18.48 (95% CI: 16.62, 20.37). Since 1995, the trend of the incidence for both sexes had been in a state of decline. Nevertheless, an upward trend remained evident on an annual basis, with parallel raised in incidence and DALYs being observed (Supplementary Table S3). From 1999 to 2021, the disease mortality trend for females (1999–2005 APC = −3.25, 95%CI: −4.11, −2.38; 2005–2021 APC = 1.59, 95%CI: 1.41,1.78) and males (2000–2006 APC = −0.64, 95%CI: −1.15, −0.13; 2006–2012 APC = 3.11, 95%CI: 2.62, 3.61; 2012–2021 APC =1.65, 95%CI:1.41, 1.88).The data demonstrated fluctuations, yet overall growth remained gradual (Figures 3C, 4C). Concurrently, the disease mortality trend of both males and females had exhibited asynchronous fluctuations, with a more pronounced downward trend observed among females. It was noteworthy that, although the age-standardized rates had exhibited an upward trend, the ASPR (AAPC = 6.43, 95% CI: 5.90, 6.96) had undergone the most significant increase.

**Table 3:** Joinpoint regression analysis: trends in age-standardized incidence and mortality rates (per 100,000 persons) among both sexes, males, and females in China, 1990–2021.

Gender		ASIR			ASMR	
	Period	APC (95% CI)	AAPC (95% CI)	Period	APC (95% CI)	AAPC (95% CI)
Both	1990–1992	1.48 (−4.30–7.61)	4.74 (4.18–5.29)*	1990–1992	1.39 (−8.39–12.22)	3.91 (2.79–5.05)*
	1992–1995	21.14 (16.55–25.92)*		1992–1995	20.44 (12.56–28.88)*	
	1995–1999	10.35 (9.00–11.73)*		1995–1998	10.81 (6.43–15.38)*	
	1999–2005	−0.77 (−1.32 to −0.22)*		1998–2001	2.88 (−1.96–7.95)	
	2005–2011	3.76 (3.26–4.26)*		2001–2004	−3.80 (−7.74–0.31)	
	2011–2021	2.64 (2.44–2.85)*		2004–2021	1.95 (1.80–2.10)*	
Female	1990–1992	1.64 (−7.47–11.63)	4.09 (3.08–5.11)*	1990–1992	1.17 (−9.12–12.61)	3.36 (2.36–4.37)*
	1992–1995	21.21 (13.74–29.16)*		1992–1995	21.59 (13.05–30.77)*	
	1995–1998	11.02 (7.08–15.10)*		1995–1999	9.38 (7.13–11.69)*	
	1998–2001	1.58 (−2.28–5.59)		1999–2005	−3.25 (−4.11 to −2.38)*	
	2001–2004	−4.46 (−7.69 to −1.13)*		2005–2021	1.59 (1.41–1.78)*	
	2004–2021	2.44 (2.29–2.59)*				
Male	1990–1992	2.88 (−1.80–7.79)	5.18 (4.79–5.58)*	1990–1992	2.55 (−3.16–8.60)	4.25 (3.77–4.74)*
	1992–1995	18.48 (16.62–20.37)*		1992–1996	18.04 (15.72–20.40)*	
	1995–1999	7.92 (6.73–9.12)*		1996–2000	7.37 (5.91–8.85)*	
	1999–2005	0.03 (−0.55–0.61)		2000–2006	−0.64 (−1.15 to −0.13)*	
	2005–2012	4.15 (3.84–4.46)*		2006–2012	3.11 (2.62–3.61)*	
	2012–2021	2.70 (2.51–2.90)*		2012–2021	1.65 (1.41–1.88)*	
						

AAPC, average annual percent change presented for full period; APC, annual percent change; CI, confidence interval.*p < 0.05.

### Age-period-cohort modeling analysis

**Figure 5:**
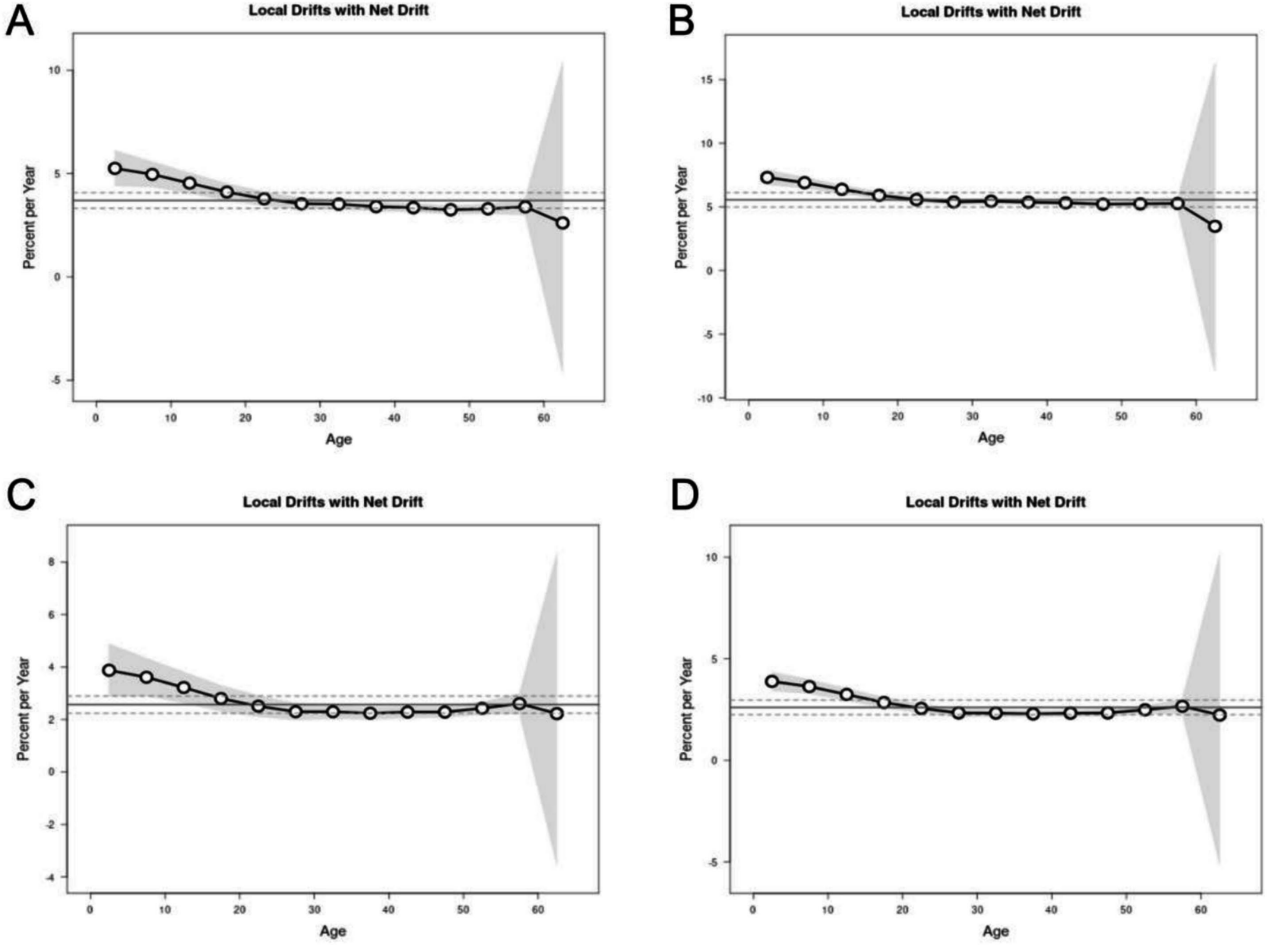
Local drifts of incidence, prevalence, mortality and DALYs for multiple myeloma in China. Age group-specific annual percent change (%) in incidence, prevalence, mortality and DALYs rate and the corresponding 95% CI. **(A)** Local drifts of incidence for multiple myeloma in China. **(B)** Local drifts of prevalence for multiple myeloma in China. **(C)** Local drifts of mortality for multiple myeloma in China. **(D)** Local drifts of DALYs for multiple myeloma in China.

The apc model results showed that the net drift of the overall annual percentage change was 3.70% (95% CI: 3.32, 4.08%) of the annual incidence, 5.55% (95% CI: 4.99, 6.12%) of the annual prevalence, 2.57% (95% CI: 2.24, 2.89%) of the annual mortality, and 2.60% (95% CI: 2.24, 2.97%) of the annual DALYs. Figure 5 shows the local drift representing the annual percentage change for each age group. The local deviation values of incidence and mortality rates in all age groups were higher than 0 (Figure 5), indicating a steady upward trend.

**Figure 6:**
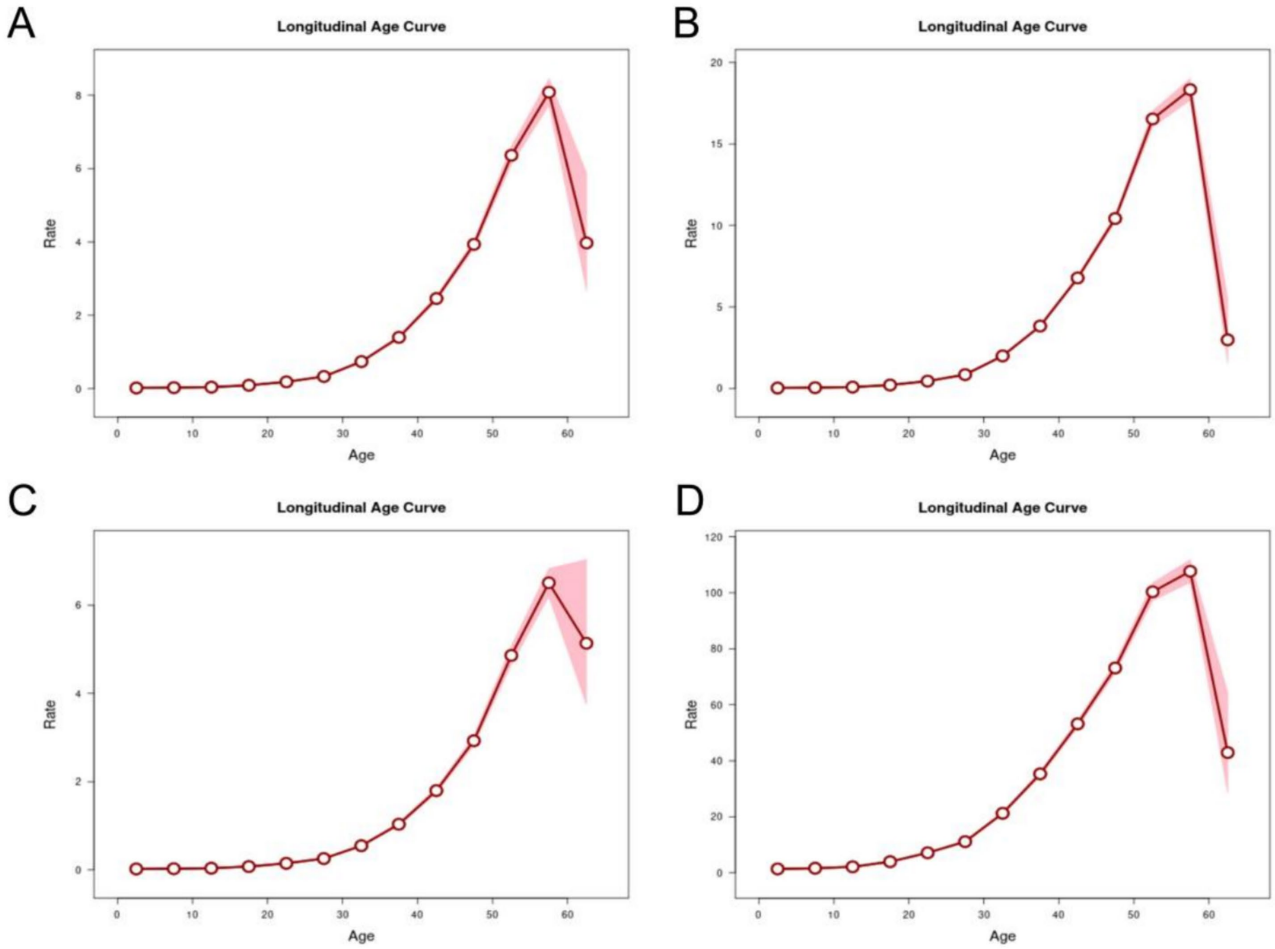
Longitudinal age curves of incidence, prevalence, mortality and DALYs for multiple myeloma in China. Fitted longitudinal age-specific rates of incidence, prevalence, mortality and DALYs (per 100,000 person-years) and the corresponding 95% CI. **(A)** Longitudinal age curves of incidence for multiple myeloma in China. **(B)** Longitudinal age curves of prevalence for multiple myeloma in China. **(C)** Longitudinal age curves of mortality for multiple myeloma in China. **(D)** Longitudinal age curves of DALYs for multiple myeloma in China.

After controlling for the effects of period and birth cohort, age effects showed that the overall risk of onset and mortality of MM in both gender populations in China from 1990 to 2021 continued to increase with age, reaching its peak in the 55–59 age group (Figure 6).

**Figure 7:**
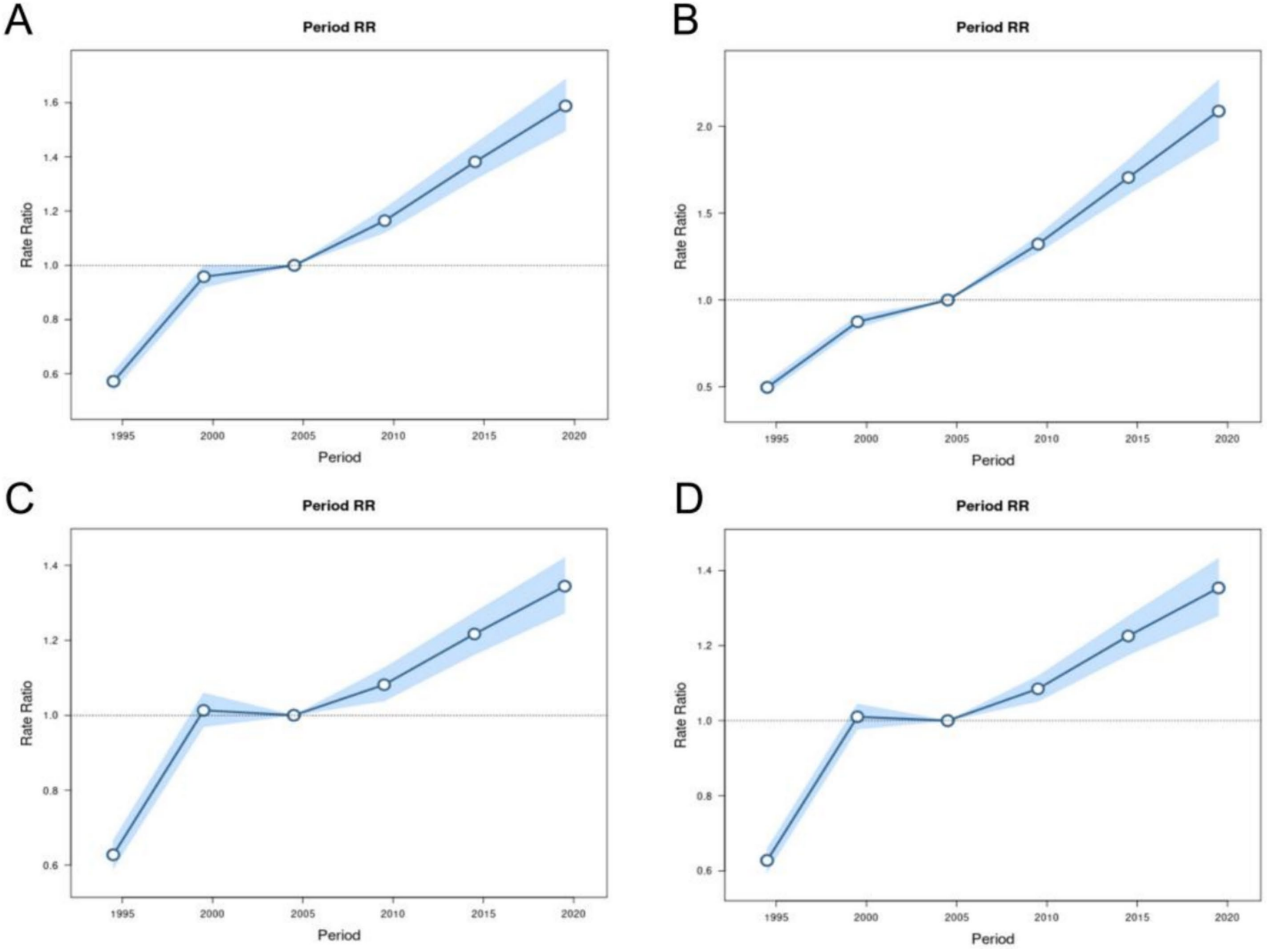
Period RRs of incidence, prevalence, mortality and DALYs for multiple myeloma in China. The RR of each period adjusted for age and non-linear cohort effects and the corresponding 95% CI. **(A)** Period RRs of incidence for multiple myeloma in China. **(B)** Period RRs of prevalence for multiple myeloma in China. **(C)** Period RRs of mortality for multiple myeloma in China. **(D)** Period RRs of DALYs for multiple myeloma in China.

After controlling for age and birth cohort effects, the period effect showed that from 1990–2021, the risk of incidence and prevalence of MM in both males and females in China showed a stable upward trend with the increase of the period, while the risk of mortality and DALYs showed a trend of first increasing, then stabilizing, and then decreasing over time. For example, the trends of mortality rate changed, taking 2004 RR = 1.00 (95% CI: 1.00, 1.00) as a reference, 1994 RR = 0.63 (95% CI: 0.59, 0.67), 1999 RR = 1.01 (95% CI: 0.97, 1.06), 2009 RR = 1.08 (95% CI: 1.04, 1.13), 2014 RR = 1.22 (95% CI: 1.16, 1.27), 2019 RR = 1.34 (95% CI: 1.27, 1.42) (Figure 7).

**Figure 8:**
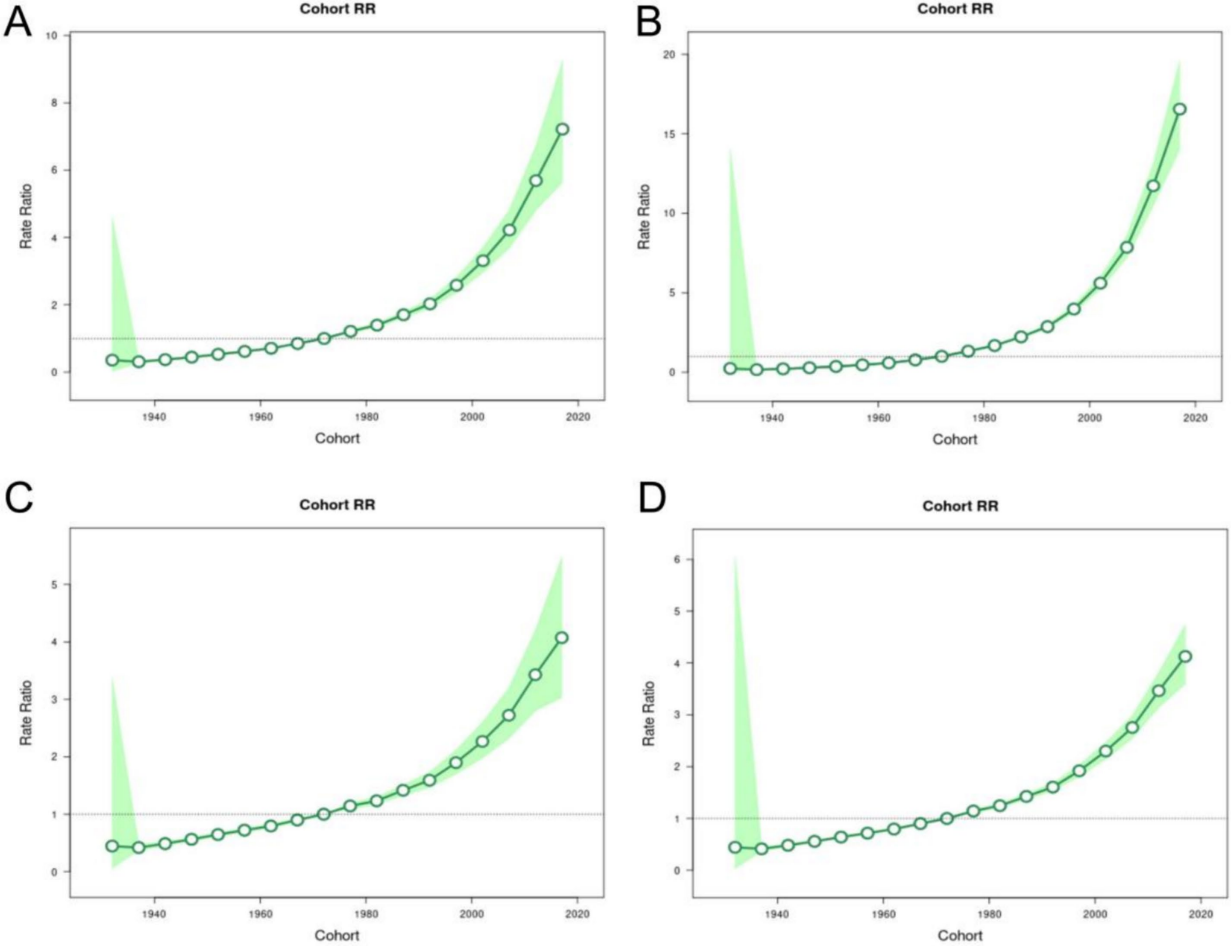
Birth cohort RRs of incidence, prevalence, mortality and DALYs for multiple myeloma in China. The RR of each birth cohort adjusted for age and non-linear period effects. **(A)** Birth cohort RRs of incidence for multiple myeloma in China. **(B)** Birth cohort RRs of prevalence for multiple myeloma in China. **(C)** Birth cohort RRs of mortality for multiple myeloma in China. **(D)** Birth cohort RRs of DALYs for multiple myeloma in China.

After controlling for age and period effects, the cohort effect showed that the incidence of MM in both genders continued to increase. Using the 1967–1972 cohort as the reference group, the risk of developing the disease reached its peak in 2012–2017 and sharply increased after 2007 (Figure 8).

Finally, according to Wald’s test results, the net drift and local drift of incidence, prevalence and DALYs rates were statistically significant (all *p < 0.001*), and the net drift of mortality was statistically significant (all *p < 0.001*), but the change of local drift was not significant (*p = 0.135*). Incidence, prevalence, mortality rates and birth cohort and period RR of DALYs were also statistically significant (all *p < 0.001*).

## Discussion

Our study comprehensively investigated the trend of disease burden in MM using the latest data (GBD 2021) from the database.

MM is the second most prevalent blood cancer subsequent to leukemia, and it manifests as a significant heterogeneity in its clinical presentation, genetic composition, and response to treatment (9). Despite the advent of novel therapeutic interventions, a proportion of patients continue to experience relapses and exhibit an unfavorable prognosis (10). The findings of this study indicated that from 1990 to 2021, ASIR, ASMR, ASPR and ASDR of MM in China increased by 4.74 (95% CI: 4.18, 5.29), 3.91 (95% CI: 2.79, 5.05), 6.43 (95% CI: 5.90, 6.96) and 3.89 (95% CI: 3.31, 4.46), respectively. Consequently, it was imperative to comprehensively assess the disease burden of MM in China.

The prevalence of MM was found to be significantly higher among males than females in China. This finding was consistent with previous research that had identified gender-specific variability in disease manifestation (11, 12). Numerous hypotheses had been postulated to explain this gender disparity. Research had indicated a modest positive correlation between higher mortality rates in males and obesity, per capita GDP, and human development index (4). However, this correlation was not statistically significant when adjusted for confounding variables such as smoking, alcohol consumption, physical inactivity, hypertension, and other potential risk factors. This finding might contradict previous views. Furthermore, the impact of sex hormones on gender disparities in disease burden required further investigation. The immunomodulatory effect of estrogen in females (13) had been suggested as a potential mechanism underlying the relationship between estrogen and MM. Some studies proposed that estrogen enhanced the immunosuppressive activity of MDSCs (14), while others indicated that estrogen could regulate the occurrence of bone disease in myeloma (15). Consequently, further exploration was necessary to elucidate the relationship between sex hormones and the pathogenesis of MM. Therefore, further exploration was necessary to elucidate the association between sex hormones and the pathogenesis of MM.

The most significant factor influencing the burden of MM in China, in terms of age, period, and birth cohort effects, was the age effect factor. This factor could be defined as the impact of changes in outcome risk associated with different age groups. In 2020, the global incidence rate of MM is concentrated among men aged 50 and above, with the highest increase in incidence rate in Germany, Denmark and South Korea. Concurrently, Denmark has the second-highest growth rate of incidence among women aged 50 and above (4). Our findings suggest that the incidence of MM before the age of 25 is extremely rare, with a higher concentration occurring after the age of 55, which is consistent with global epidemiological trends. The incidence and mortality rates of MM continue to rise with age, indicating that the risk of incidence and mortality in the older adult population cannot be overlooked. Despite the positive impact of improved diagnostic and treatment techniques on the mortality rate of MM and significant changes in the treatment pattern, the mortality rate of MM in China is still rising steadily. The following factors may be contributing to this trend. Firstly, the aging population is a key factor driving the increase in the disease burden of MM in China (16). Secondly, the proportion of older adult people in developed provinces or urban areas in China is higher and the aging trend is more obvious, which will essentially increase the incidence rate and mortality of MM. This geographical difference cannot be ignored (17). Thirdly, while the overall mortality rate of MM worldwide has shown a downward trend, there are significant differences between countries, and the degree of decline in mortality seems to be related to age at diagnosis. The elders are weakened due to comorbidities, poor physical or cognitive function, which complicates the management of MM (18) and increases the mortality burden of MM in the elders compared to the younger adults (19). Furthermore, the treatment outcomes of the elders with MM are often influenced by comorbidities and an increased susceptibility to treatment adverse events. The older adult population is characterized by heterogeneity, with an elevated prevalence of frailty (defined as a state of weakness and vulnerability that exceeds that observed in other age groups) (5). Consequently, this segment of the figure is set to expand, owing to the substantial population base of China. This has resulted in a steady rise in the incidence and mortality rates of the country in recent years.

The results of the period effect demonstrated that the risk of MM increases over time, and the overall risk of death shows a gradual upward trend. This rising trend may be attributable to enhanced disease diagnosis and an increase in environmental and metabolic risk factors. The period 1992–1996 saw a deepening of China’s medical reform, improvements in the diagnosis and treatment of MM, and the emergence of new detection platforms such as multi-parameter flow cytometry (20). At the same time, there was an update in early screening, comprehensive diagnosis and detection technology for MM (21). This may provide an explanation for the increase in the incidence rate of MM. Concurrently, China’s advancement in medical care services and enhancement in cancer screening has facilitated access to timely diagnoses and treatments for individuals. Consequently, the incidence rate and mortality of men and women in this period have increased significantly. Geographical differences between provinces are also a significant factor in the epidemiological characteristics of MM in China. The proportion of the older adult in developed provinces or urban areas of China is high and the aging trend is more obvious, which will increase the incidence rate and mortality of MM in essence. This is not synchronized with the overall downward trend in the mortality rate of MM worldwide (4). It is important to note that the issue of aging may become a significant medical challenge in China due to its large population base (22).

The queue effect was widely considered to reflect exposure to specific risk factors during early life, which were not present during other periods. The significance of studying the queue effect lied in the fact that queues represent the most natural way of gathering individuals, as the prevalence of etiological factors related to the environment or lifestyle is largely determined by the year of birth. In the present study, it was found that due to economic and medical limitations in the early years, individuals in the early birth cohort were unable to receive timely diagnosis and treatment. The social and medical conditions prevalent in China at that time were also a contributing factor. The awareness of health knowledge among residents was low, and unhealthy lifestyles such as smoking, excessive alcohol consumption, lack of exercise, and unreasonable diet were common (23). Concurrently, individuals experienced heightened exposure to diverse physical and chemical agents, including ionizing radiation. Consequently, the risk of onset and mortality was higher in early and mid birth cohorts. In comparison with earlier cohorts, the late-born cohort received a robust education (24), developed a renewed understanding of disease and health, and experienced a period of rapid medical development, resulting in fluctuations in the risk of prevalence and mortality.

The data for this study was sourced from the GBD 2021 database, which was widely regarded as a reliable source of research data. The database was currently regarded as one of the most standardized and accurate systems for evaluating the burden of related diseases. Its scope was extensive, encompassing a wide timeframe, diverse geographical regions, and a comprehensive range of diseases and injuries. Furthermore, the absence of China from the database was a notable limitation, as it precluded the possibility of conducting further analysis due to the unavailability of regional data. This study employed a comprehensive time frame for analysis and encompasses a diverse range of populations. The study collected data on incidence, mortality and DALYs of MM in different gender and age groups over a 30-year period, and analyzed their long-term trends. This study addressed a significant gap in the long-term trend of the burden of MM disease in China.

In summary, the ASIR and ASMR of Chinese MM showed a significant upward trend from 1990 to 2021, with the increase in ASPR being the most significant. Another notable finding is that MM is more common in males than in females. The apc model suggests that with increasing age, the older adult population becomes a high-risk group. In the context of an ageing and growing population, increased surveillance and better disease control are needed to reduce the burden of MM.

## Conclusion

To conclude, the burden of MM in China was studied using the latest data from GBD 2021. The incidence and mortality had increased markedly in males and the elders. Although the current prevalence of MM in China was low compared to global levels, the increasing incidence must be taken seriously. The apc model highlighted the importance of age, period and cohort factors in understanding the epidemiology of MM. The results of this study will help to emphasize decision-making in MM disease prevention and treatment programming to minimize the burden of MM due to the large population base and aging trends in China.

## Data Availability

The original contributions presented in the study are included in the article/Supplementary material, further inquiries can be directed to the corresponding authors.

## Footnotes

^1^ https://ghdx.healthdata.org/gbd-2021
