# Supplementary material for: Long-term trends in the burden of multiple myeloma in China: a Joinpoint regression and age-period-cohort analysis based on GBD 2021
Source: Front Public Health. 2025 Feb 12;13:1554485. doi: 10.3389/fpubh.2025.1554485 (PMC11861083; doi:10.3389/fpubh.2025.1554485)

Supplementary Figures

Supplementary Figure 1. Age-specific numbers and age-standardized YLDs and YLLs rates of multiple myeloma in China, 2021. (A) Age-specific YLDs number. (B) Age-standardized YLDs rate. (C) Age-specific YLLs number. (D) Age-standardized YLLs rate.


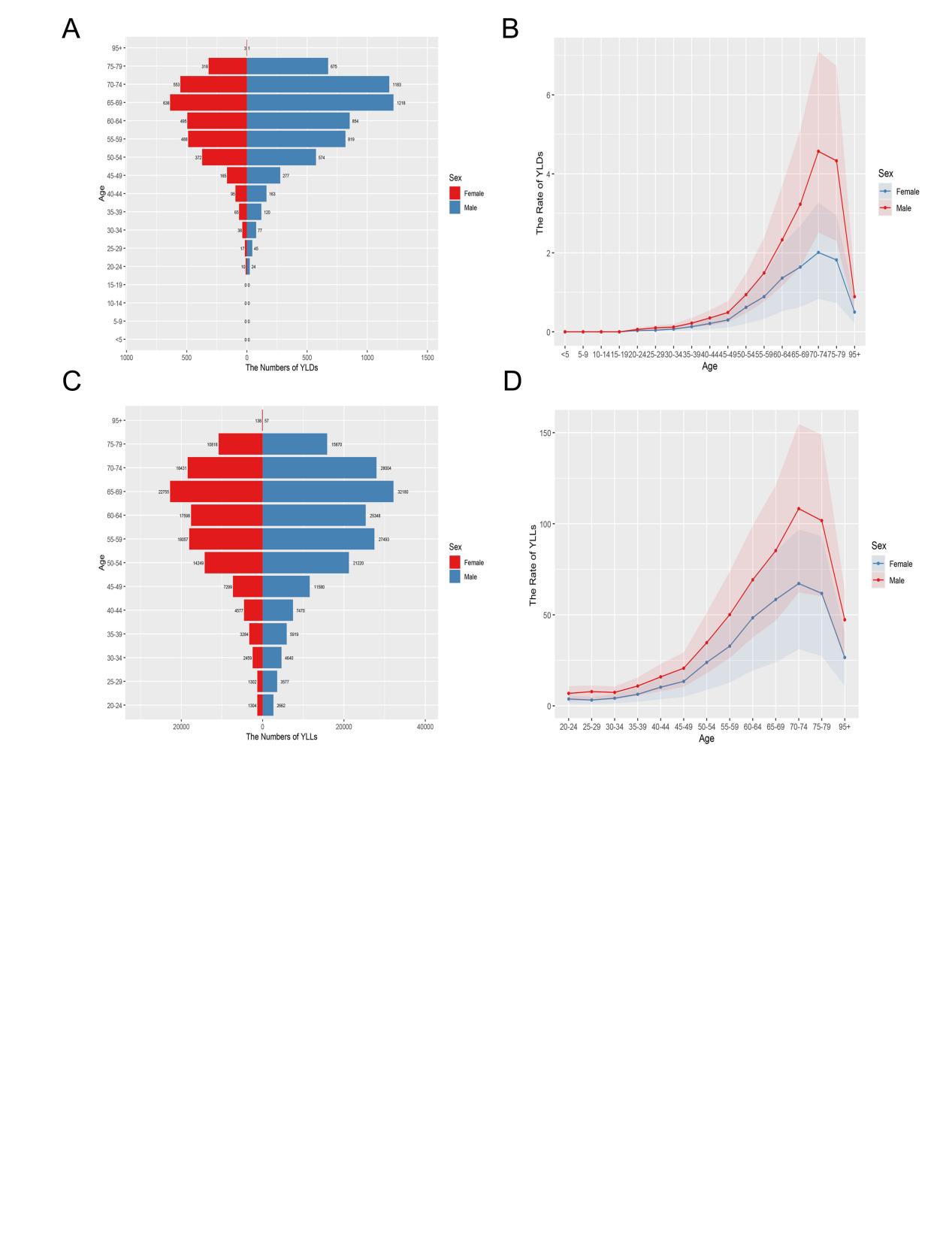


Supplementary Figure 2. Trends in the all-age cases and age-standardized YLDs and YLLs rates of multiple myeloma by sex from 1990-2021. (A) YLDs number and rate. (B) YLLs number and rate.


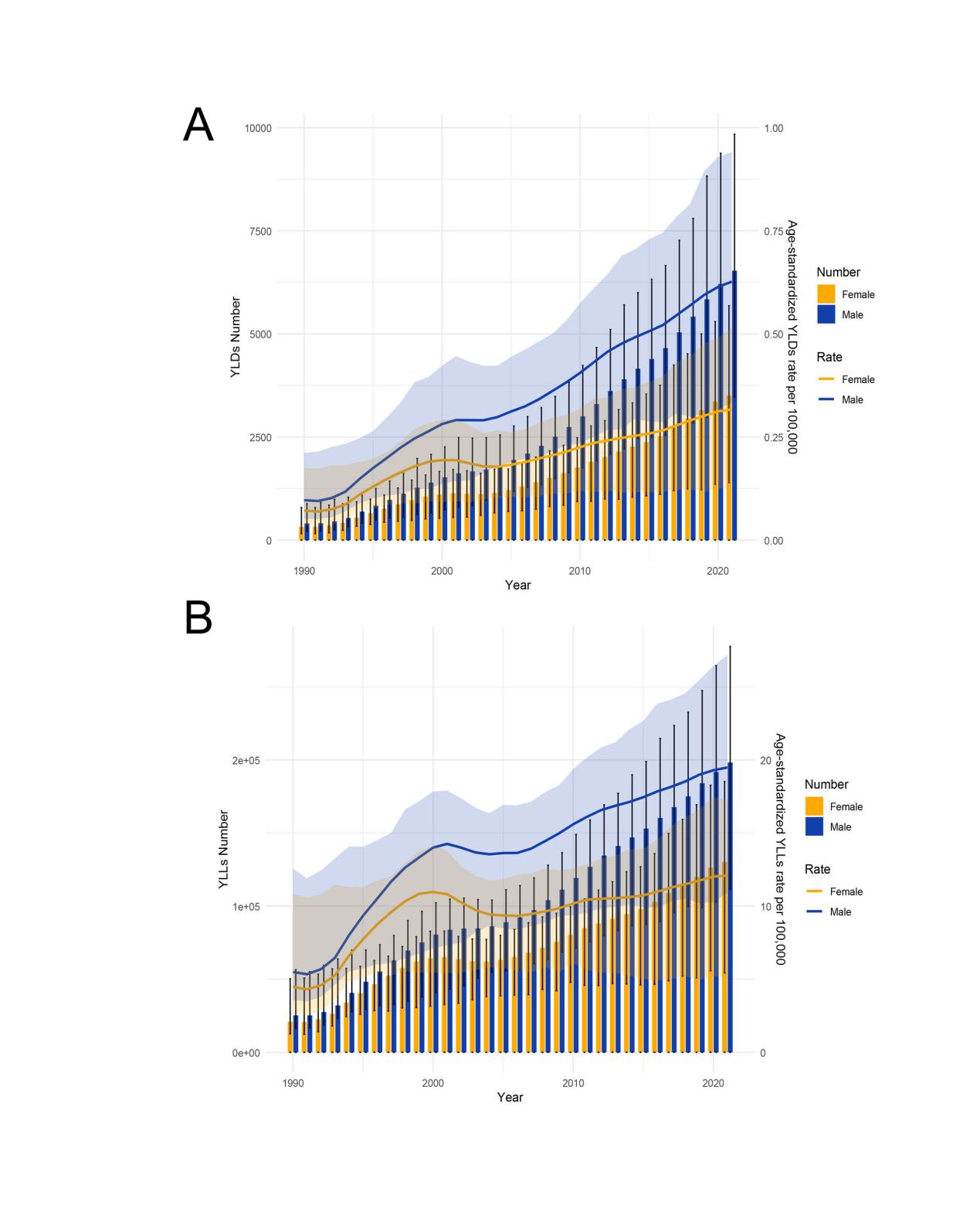

Supplement: Supplementary file 1 [file Supplementary_file_1.docx]
