# Supplementary material for: Long-term trends in the burden of multiple myeloma in China: a Joinpoint regression and age-period-cohort analysis based on GBD 2021
Source: Front Public Health. 2025 Feb 12;13:1554485. doi: 10.3389/fpubh.2025.1554485 (PMC11861083; doi:10.3389/fpubh.2025.1554485)
Supplement: Supplementary file 2 [file Table_1.docx]

Supplementary Table 1. All-age cases prevalence, incidence, deaths, DALYs, YLDs and YLLs rates and change in 1990 and 2021 for multiple myeloma in China.

| Measure | All-ages cases | | |
| --- | --- | --- | --- |
|  | 1990 | 2021 | Change |
|  | n (95% CI) | n (95% CI) |  |
| Prevalence | 2977 (2052,5851) | 47004 (29544,62136) | 1.48% |
| Incidence | 1693 (1154,3360) | 17250 (11017,22663) | 9.19% |
| Deaths | 1591 (1080,3159) | 12984 (8448,17114) | 7.16% |
| DALYs | 46854 (32056,92593) | 338359 (213669,447635) | 6.22% |
| YLDs | 725 (400,1489) | 10036 (6091,14597) | 1.28% |
| YLLs | 46129 (31608,91267) | 328323 (207346,433763) | 6.12% |

Rate of change = (2021indicator value - 1990 indicator value)/1990 indicator value × 100 percent;CI, confidence interval

Supplementary Table 2. Age-standardized prevalence, incidence, deaths, DALYs, YLDs and YLLs rates and change in 1990 and 2021 for multiple myeloma in China.

| Measure | Age-standardized rates per 100 000 people | | |
| --- | --- | --- | --- |
|  | 1990 | 2021 | Change |
|  | n (95% CI) | n (95% CI) |  |
| Prevalence | 0.32 (0.22,0.64) | 2.19 (1.37,2.9) | 5.84% |
| Incidence | 0.20 (0.13,0.39) | 0.81 (0.52,1.07) | 3.05% |
| Deaths | 0.19 (0.13,0.39) | 0.62 (0.40,0.81) | 2.26% |
| DALYs | 5.02 (3.42,9.99) | 16.12 (10.09,21.35) | 2.21% |
| YLDs | 0.08 (0.05,0.17) | 0.46 (0.28,0.67) | 4.75% |
| YLLs | 4.94 (3.36,9.82) | 15.66 (9.80,20.70) | 2.17% |

Rate of change = (2021indicator value - 1990 indicator value)/1990 indicator value × 100 percent;CI, confidence interval

Supplementary Table 3 Joinpoint regression analysis: trends in age-standardized prevalence and DALYs rates (per 100,000 persons) among both sexes, males, and females in China, 1990–2021.

| Gender | ASPR | | | ASDR | | |
| --- | --- | --- | --- | --- | --- | --- |
|  | Period | APC (95% CI) | AAPC (95% CI) | Period | APC (95% CI) | AAPC (95% CI) |
| Both | 1990-1992 | 2.16 (-3.26 - 7.89) | 6.43 (5.90 - 6.96)* | 1990-1992 | 0.88 (-5.09 - 7.23) | 3.89 (3.31 - 4.46)* |
|  | 1992-1995 | 22.12 (17.84 - 26.56)* |  | 1992-1995 | 20.36 (15.63 - 25.28)* |  |
|  | 1995-1999 | 11.43 (10.08 - 12.79)* |  | 1995-1999 | 9.57 (8.13 - 11.03)* |  |
|  | 1999-2004 | 0.66 (-0.13 - 1.46) |  | 1999-2006 | -1.42 (-1.85 - -0.99)* |  |
|  | 2004-2012 | 6.01 (5.71 - 6.31)* |  | 2006-2011 | 3.24 (2.49 - 3.99)* |  |
|  | 2012-2021 | 4.05 (3.80 - 4.30)* |  | 2011-2021 | 1.85 (1.63 - 2.07)* |  |
| Female | 1990-1992 | 1.91 (-4.26 - 8.48) | 5.60 (5.00 - 6.20)* | 1990-1992 | 0.79 (-7.03 - 9.27) | 3.34 (2.59 - 4.11)* |
|  | 1992-1995 | 22.58 (17.51 - 27.86)* |  | 1992-1995 | 21.00 (14.49 - 27.88)* |  |
|  | 1995-1999 | 10.70 (9.33 - 12.08)* |  | 1995-1999 | 9.12 (7.42 - 10.86)* |  |
|  | 1999-2004 | -1.45 (-2.24 - -0.65)* |  | 1999-2005 | -3.16 (-3.84 - -2.49)* |  |
|  | 2004-2011 | 5.22 (4.79 - 5.64)* |  | 2005-2021 | 1.74 (1.60 - 1.88)* |  |
|  | 2011-2021 | 3.57 (3.32 - 3.82)* |  |  |  |  |
| Male | 1990-1992 | 3.71 (-1.57 - 9.27) | 6.99 (6.52 - 7.46)* | 1990-1992 | 2.36 (-2.84 - 7.84) | 4.30 (3.86 - 4.75)* |
|  | 1992-1996 | 19.67 (17.56 - 21.82)* |  | 1992-1996 | 17.69 (15.59 - 19.81)* |  |
|  | 1996-2000 | 9.04 (7.66 - 10.43)* |  | 1996-2000 | 7.03 (5.70 - 8.38)* |  |
|  | 2000-2004 | 1.52 (0.39 - 2.67)* |  | 2000-2006 | -0.83 (-1.31 - -0.34)* |  |
|  | 2004-2012 | 6.59 (6.29 - 6.88)* |  | 2006-2011 | 3.81 (3.15 - 4.48)* |  |
|  | 2012-2021 | 4.38 (4.15 - 4.62)* |  | 2011-2021 | 2.01 (1.82 - 2.20)* |  |

AAPC, average annual percent change presented for full period; APC, annual percent change; CI, confidence interval.*P<0.05.

Supplementary Table 4. The incidence, prevalence, mortality and DALYs relative risks of multiple myeloma in China due to period and birth cohort effects.

| Factor | Incidence (RR,95%CI) | Prevalence (RR,95%CI) | Mortality (RR,95%CI) | DALYs (RR,95%CI) |
| --- | --- | --- | --- | --- |
| Period RR |  |  |  |  |
| 1994 | 0.57 (0.54, 0.61) | 0.50 (0.47, 0.53) | 0.63 (0.59, 0.67) | 0.63 (0.60, 0.66) |
| 1999 | 0.96 (0.92,1.00) | 0.87 (0.84, 0.91) | 1.01 (0.97, 1.06) | 1.01 (0.98, 1.04) |
| 2004 | 1.00 (1.00, 1.00) | 1.00 (1.00, 1.00) | 1.00 (1.00, 1.00) | 1.00 (1.00, 1.00) |
| 2009 | 1.16 (1.12, 1.21) | 1.32 (1.27, 1.37) | 1.08 (1.04, 1.13) | 1.08 (1.05, 1.12) |
| 2014 | 1.38 (1.32, 1.45) | 1.71 (1.61, 1.81) | 1.22 (1.16, 1.27) | 1.23 (1.18, 1.28) |
| 2019 | 1.59 (1.50. 1.69) | 2.09 (1.92, 2.27) | 1.34 (1.27, 1.42) | 1.35 (1.28, 1.43) |
| Birth Cohort RR |  |  |  |  |
| 1927-1932 | 0.36 (0.03, 4.59) | 0.24 (0.00, 14.01) | 0.45 (0.06, 3.36) | 0.44 (0.03, 6.02) |
| 1932-1937 | 0.31 (0.27, 0.35) | 0.16 (0.14, 0.19) | 0.42 (0.37, 0.47) | 0.41 (0.37, 0.46) |
| 1937-1942 | 0.37 (0.35, 0.40) | 0.21 (0.20, 0.23) | 0.49 (0.45, 0.53) | 0.48 (0.45, 0.51) |
| 1942-1947 | 0.45 (0.42, 0.47) | 0.28 (0.27, 0.29) | 0.56 (0.53, 0.60) | 0.56 (0.53, 0.58) |
| 1947-1952 | 0.53 (0.51, 0.56) | 0.36 (0.35, 0.38) | 0.65 (0.61, 0.68) | 0.64 (0.62, 0.67) |
| 1952-1957 | 0.62 (0.59, 0.64) | 0.46 (0.45, 0.48) | 0.72 (0.69, 0.76) | 0.72 (0.69, 0.74) |
| 1957-1962 | 0.71 (0.68, 0.74) | 0.59 (0.57, 0.60) | 0.79 (0.76, 0.83) | 0.80 (0.77, 0.82) |
| 1962-1967 | 0.85 (0.82, 0.88) | 0.78 (0.76, 0.80) | 0.90 (0.86, 0.94) | 0.90 (0.88, 0.93) |
| 1967-1972 | 1.00 (1.00, 1.00) | 1.00 (1.00, 1.00) | 1.00 (1.00, 1.00) | 1.00 (1.00, 1.00) |
| 1972-1977 | 1.21 (1.17, 1.26) | 1.33 (1.29, 1.36) | 1.14 (1.09, 1.20) | 1.14 (1.11, 1.18) |
| 1977-1982 | 1.40 (1.33, 1.46) | 1.68 (1.63, 1.73) | 1.23 (1.17, 1.30) | 1.25 (1.21, 1.29) |
| 1982-1987 | 1.70 (1.61, 1.80) | 2.22 (2.14, 2.31) | 1.42 (1.33, 1.51) | 1.43 (1.37, 1.48) |
| 1987-1992 | 2.03 (1.89, 2.18) | 2.87 (2.74, 3.01) | 1.59 (1.46, 1.73) | 1.61 (1.53, 1.68) |
| 1992-1997 | 2.58 (2.36, 2.82) | 3.98 (3.75, 4.22) | 1.90 (1.70, 2.12) | 1.92 (1.81, 2.04) |
| 1997-2002 | 3.31 (2.96, 3.70) | 5.61 (5.21, 6.04) | 2.27 (1.98, 2.60) | 2.30 (2.14, 2.47) |
| 2002-2007 | 4.22 (3.69, 4.83) | 7.86 (7.19, 8.60) | 2.72 (2.32, 3.20) | 2.76 (2.54, 2.99) |
| 2007-2012 | 5.69 (4.80, 6.73) | 11.73 (10.47, 13.14) | 3.43 (2.80, 4.20) | 3.46 (3.14, 3.82) |
| 2012-2017 | 7.22 (5.63, 9.24) | 16.56 (14.01, 19.58) | 4.07 (3.03, 5.47) | 4.13 (3.59, 4.74) |

Abbreviations: RR, relative risks; CI, confidence interval; DALYs, disability-adjusted life years.
